# DEP-Dots for 3D cell culture: low-cost, high-repeatability, effective 3D cell culture in multiple gel systems

**DOI:** 10.1038/s41598-020-71265-7

**Published:** 2020-09-03

**Authors:** Erin A. Henslee, Carina M. Dunlop, Christine M. de Mel, Emily A. Carter, Rula G. Abdallat, Patrizia Camelliti, Fatima H. Labeed

**Affiliations:** 1 Centre for Biomedical Engineering, Department of Mechanical Engineering Sciences, University of Surrey, Guildford, GU2 7XH Surrey UK; 2 Department of Mathematics, University of Surrey, Guildford, GU2 7XH Surrey UK; 3 School of Biosciences and Medicine, University of Surrey, Guildford, GU2 7XH Surrey UK; 4 Present Address: Department of Engineering, Wake Forest University, Wake Downtown, Winston-Salem, NC 27109 USA; 5 Present Address: Department of Biomedical Engineering, Faculty of Engineering, The Hashemite University, PO Box 330127, Zarqa, 13133 Jordan

**Keywords:** Biophysics, Cancer, Computational biology and bioinformatics, Drug discovery, Engineering, Mathematics and computing

## Abstract

It is known that cells grown in 3D are more tolerant to drug treatment than those grown in dispersion, but the mechanism for this is still not clear; cells grown in 3D have opportunities to develop inter-cell communication, but are also closely packed which may impede diffusion. In this study we examine methods for dielectrophoresis-based cell aggregation of both suspension and adherent cell lines, and compare the effect of various drugs on cells grown in 3D and 2D. Comparing viability of pharmacological interventions on 3D cell clusters against both suspension cells and adherent cells grown in monolayer, as well as against a unicellular organism with no propensity for intracellular communication, we suggest that 3D aggregates of adherent cells, compared to suspension cells, show a substantially different drug response to cells grown in monolayer, which increases as the IC_50_ is approached. Further, a mathematical model of the system for each agent demonstrates that changes to drug response are due to inherent changes in the system of adherent cells from the 2D to 3D state. Finally, differences in the electrophysiological membrane properties of the adherent cell type suggest this parameter plays an important role in the differences found in the 3D drug response.

## Introduction

Whilst culture of adherent cells is most commonly performed by placing the cells in a flask and growing them in a monolayer, methods of producing three-dimensional (3D) cellular constructs have gained significant attention in recent years. For example, evidence suggests that 3D structures can be a more accurate predictive model of drug response compared to conventional two-dimensional (2D) (monolayer) models. With 90% of promising preclinical drug treatments failing during human clinical trials, 3D models have emerged to bridge the gap between in vitro and in vivo cellular drug responses, whilst circumventing the numerous challenges in throughput associated with animal models^1–6^. In vitro models can provide the large sample sizes necessary for preclinical tests, and as a consequence recent development has focused on creating 3D systems that adequately mimic living tissue to reduce the need for animal modelling. 3D models have demonstrated that maintenance of natural cell shape, cell–cell and cell-extracellular matrix (ECM) interactions, as well as the effects of tissue architecture, all affect disease progression and drug responses in cells^6–9^. For example, disease progression and drug efficacy in cancer have been shown to vary significantly between 2D versus 3D constructs^6–9^. There is also debate about whether the mechanism underpinning this observed increase in drug resistance in 3D models is due to changes in the behaviour of cells due to cell-to-cell contact, the architecture contributing to varied cell-signalling, or simply that the inner cells in a 3D construct are shielded from the drug due to the outer cells preventing diffusion of the drug across the whole cell mass, or drug diffusion parameters altering in the 3D models^4,5^.

Early systems for 3D spheroid formation such as spinners, shakers, rotaries, and the hanging drop method are simple to use and are designed for mass fabrication. However, it is difficult to use these systems whilst maintaining a uniform size and geometry in micro-scale spheroids, which are crucial when investigating avascular effects in cell constructs^9–15^. Recent technologies have improved both the ease of use and geometrical consistency in these assays and have been used for many tissue engineering applications. Techniques include Perfecta3D and InSphero’s GravityPlus which have been utilised to investigate drug treatments on cancer as well as creating micro-tissues of liver, cardiac, and skin tissues^16^, whilst the AggreWell plate which has primarily been utilised to induce various cell differentiation from stem cells^4,5,10,16–19^.

In addition to these techniques, dielectrophoresis (DEP) has been employed as a method to construct cell clusters in polymer hydrogels. DEP has been used for creating stem cell niches^20,21^ and to assess drug efficacy^22–24^. DEP is the movement of polarisable particles in a non-uniform electric field^25–27^ and causes either attraction to (positive DEP) or repulsion from (negative DEP) high electric field regions, such as electrode edges. It can be used to position cells by directing cells to move to a place of aggregation, or as a tool for assessing their electrophysiology by measuring frequency-dependent behaviour. It provides an attractive alternative technique for cell patterning; it is rapid, simple and inexpensive, and the use of electrodes to form the shape of the cell aggregate provides versatility when patterning.

In this paper we use an on-chip, DEP-dot electrode^15,28^ device as a robust, high throughput, reproducible technique of cell aggregation, and investigate the effects of 3D encapsulation on drug effectiveness for suspension and adherent cell lines. By comparing the effects of pharmacological interventions on both encapsulated 3D cell clusters against both suspension cells and adherent cells grown in their 2D environment (suspension or monolayer), we suggest that 3D aggregates of adherent cells show a substantially different drug response to cells grown in monolayer, which increases as the IC_50_ is approached, whilst monodisperse suspension cells exhibited similar drug response to those in clusters. To explore the differences found in these 2D versus 3D models further, a mathematical model of the system for each agent is described; this suggests that changes to drug response are due to inherent changes in adherent cells’ response from the 2D to 3D state. The efficacy of the dot system is also analysed using a range of gels including collagen and PuraMatrix, which enables dissociation of the cell aggregates post 3D encapsulation; we then use DEP to analyse these encapsulated cells and examine differences in the electrophysiological properties of 2D versus dissociated 3D cultured cells. Differences within the membrane properties of the adherent cell type suggest this parameter plays an important role in the differences found in the 3D drug response.

## Results and discussion

### DEP dots demonstrate robust, repeatable aggregation

**Figure 1:**
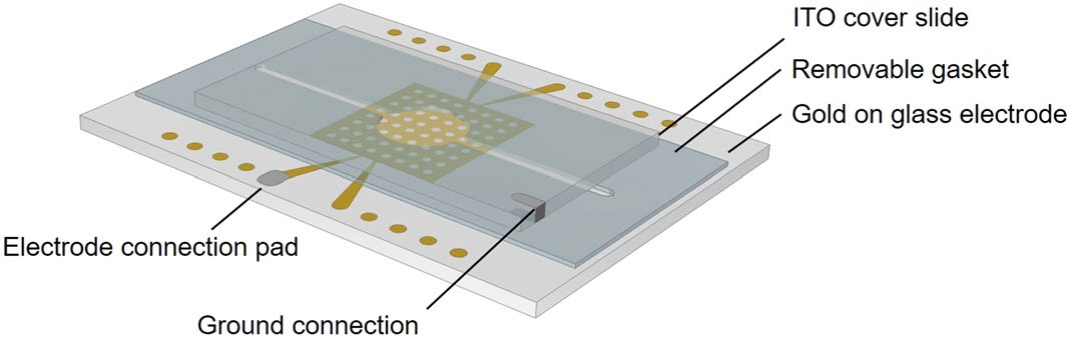
Schematic of the DEP dot array. The removable gasket connects the ITO ground electrode to the gold array and also serves as the reservoir in which the CPM and cell mixture is contained. The height of the gasket is approximately 280 µm.

Dot electrodes (Fig. 1) use negative (repulsive) DEP to contain the cells, by repelling them from the edges of circular patterns in the electrode and forcing them into aggregates^15^. Negative DEP occurs at specific frequency ranges, determined by the electrical properties of cells and medium. Optimal frequencies were established for each cell type used in the formation of aggregates using a DEPtech 3DEP reader (Labtech, Heathfield, UK) as described previously^15,22,29^. This approach allowed for cell specific aggregation protocols and control of construct size and geometry.

**Figure 2:**
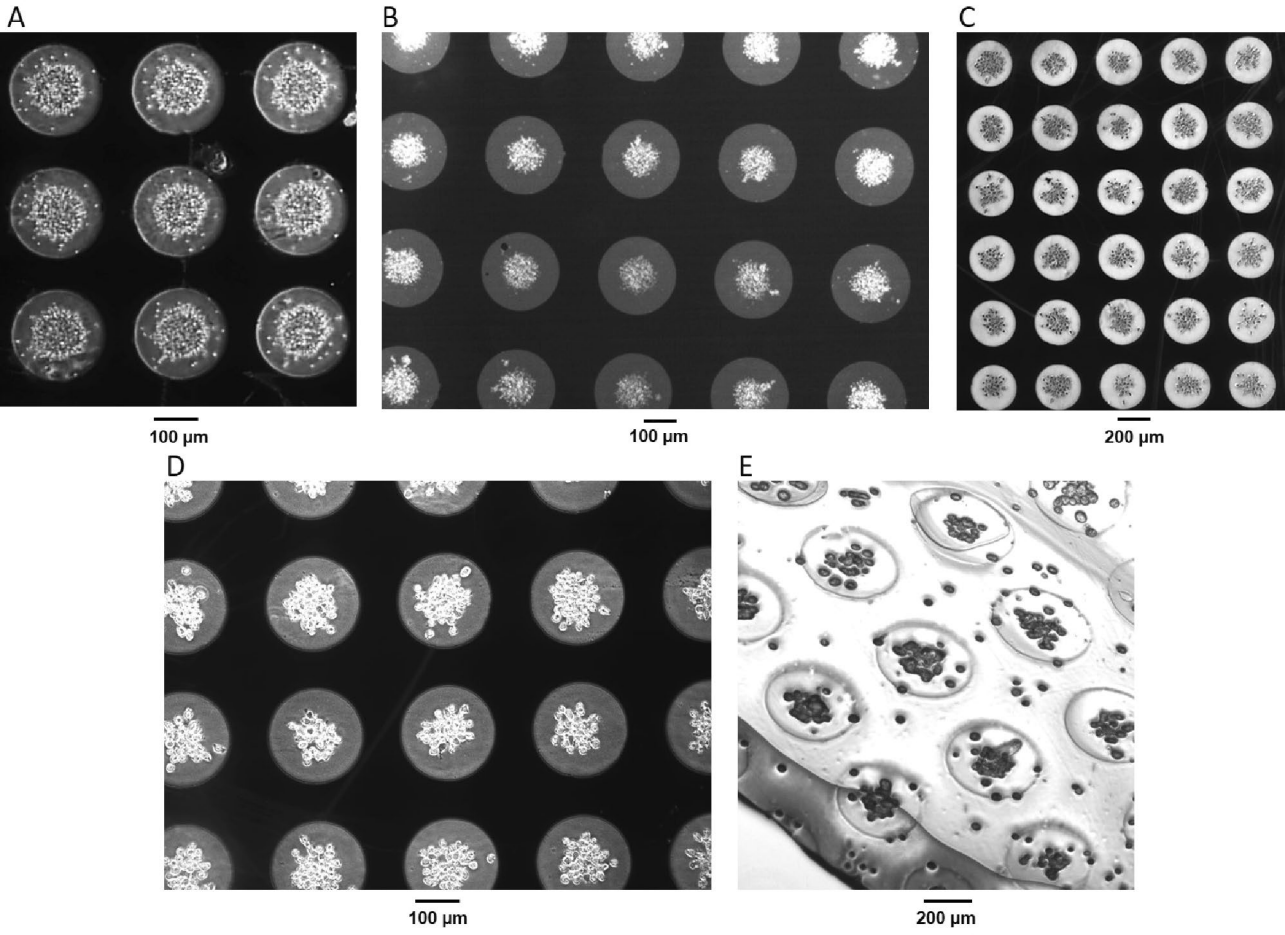
DEP DOT Electrode repeatability across cell types. The following cell types were encapsulated in PEG-DA (**A**) yeast, (**B**) HL-1 (**C**) K562, and (**D**) HeLa. The resultant peeled gel is shown (**E**) with a thickness of approximately 280 µm and width of approximately 5 mm. The peeled gels, like that shown, were placed in well plates for cell culture and subsequent drug treatment.

In order to assess the repeatability (n = 100) of aggregate formation in the polyethylene glycol diacrylate (PEG-DA) system, aggregate size and cell number were measured for different cell types with different properties and diameters, and dot radii between 100 and 150 µm. Examples of these aggregates are shown in Fig. 2. The number of cells per aggregate were measured, showing no significant difference (p > 0.5) to their respective means, for all cell types. When aggregate size was compared to the electrode size used for aggregation, all cell lines demonstrated similar (p > 0.1) aggregate size to dot size ratios (Table 1) with aggregates covering just under half of the dot diameter. Since the cell lines used were of similar radii (~ 10 µm), and resulted in similar aggregate size, this demonstrates that the platform can reliably produce aggregates of uniform size. Interestingly, though the aggregates were of similar relative size, as were the cells, the number of cells per aggregate varied, suggesting that changes in cell morphology allowed for significantly different packing densities.

**Table 1:** Aggregate characterisation (n = 100) for the various cell types.

Cell type	Frequency (kHz)	Cell radius (µm)	Cells per aggregate	Aggregate diameter/dot diameter
Yeast	10	3.5 ± 0.8	300 ± 26	0.465 ± 0.04
HeLa	5	10.3 ± 1.1	32 ± 5	0.462 ± 0.03
K562	7	10.4 ± 1.5	54 ± 8	0.468 ± 0.03
HL-1	2	10.2 ± 1.5	75 ± 6	0.456 ± 0.02

We also established the UV exposure during cross-linking did not negatively affect cell viability by monitoring viability with Trypan Blue (as described in Abdallat et al.^15^). There was no significant difference (p > 0.1) in viability values, with viabilities recorded in ranges between 92–100% for HeLa cells and 94–97% for yeast cells when comparing 2D cell culture methods and cells in 3D aggregates.

### DEP-Dots can form aggregates in multiple gelling agents

Since UV light and the photo-initiator 2,2-dimethoxy-2-phenylacetophenone (DMPA) can be hazardous to certain cells, it is beneficial for a 3D cell construction technique to be sufficiently flexible to allow the use of alternative gelling agents which are activated by different means; similarly, a user may wish to form a gel that (unlike PEG-DA) can be subsequently broken down to allow recovery of the aggregated cells. To this end we investigated multiple approaches to gel formation using the DEP-Dots.

**Figure 3:**
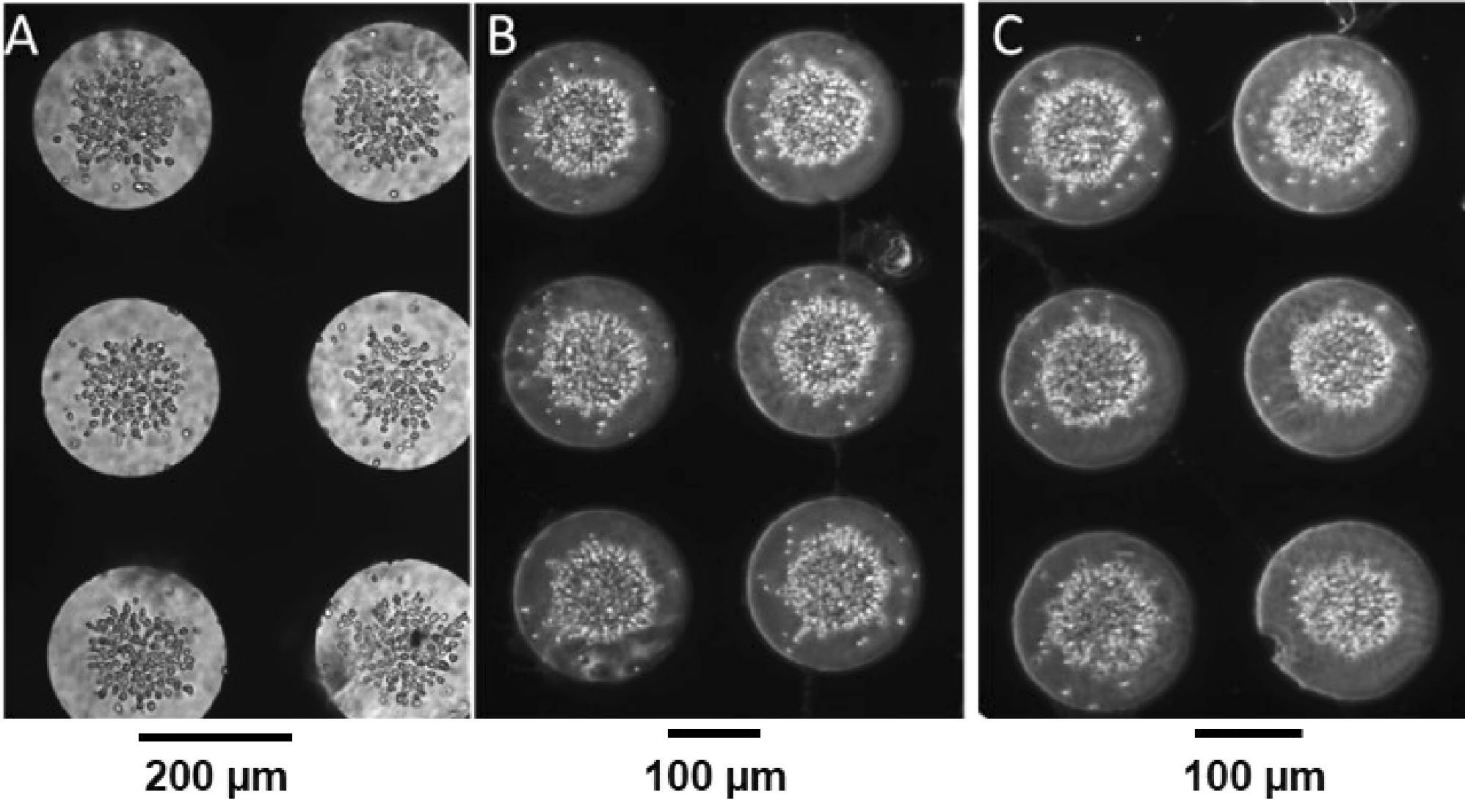
The DEP DOT Electrode system is consistent in cell aggregation using various gel systems. (**A**) K562 cells in PEG-DA cross linked under blue light with initiators TEA and eosin-Y. (**B**) Yeast cells in 6.75% w/v collagen, and (**C**) Yeast cells in 25% PuraMatrix.

First, in order to avoid the use of UV exposure we investigated a more biocompatible system using PEG-DA and the blue light initiators triethanolamine (TEA) and eosin-Y^27^ (Fig. 3A). Cells were observed to form patterns similar to those formed using the UV initiator, as described in the previous section. However, the blue light initiator required a longer curing time (over 10 min under blue light). Aggregate relative size and cell number was consistent with our previous PEG-DA experiments.

Second, we investigated gels with different functional characteristics. A distinct disadvantage of PEG-based systems is that once cells are encapsulated, they remain in the gel permanently. This does not allow for post treatment analysis of the individual cells, or layers of these cells. One particularly useful tool would be to characterize changes in the cells pre- and post-encapsulation, for example to allow analysis of cell behaviour using flow cytometry. We have previously shown these electrophysiological properties can predict drug IC_50_^22^; if cells could be obtained post-encapsulation a similar comparison could be performed.

To this end, other hydrogel options were then investigated for aggregate dissociation. Collagen hydrogels (Fig. 3B) are widely used for scaffolding and tissue engineering. However, this is a challenging gel system for use in DEP, due to the composition; the solution is highly conductive and the larger amounts necessary for a durable gel (for complete removal from the chip) increase the viscosity, rendering movement by DEP force difficult. We were able to minimize these effects within the dot array at sufficiently low gel content, and determined 6.75 w/v% with a solution conductivity of 875 mS/m achieved adequate negative DEP response, and produced a sufficiently strong gel to be peeled from the chip intact. This gel system was temperature cured, so samples were kept warmed to 40 °C, aggregated with DEP, and then placed in an incubator set to 20 °C for 10 min. These remained intact, and the cells remained viable for at least 1 week after gel formation.

PuraMatrix (Fig. 3C) was also investigated, with concentrations of 25% yielding adequate durability of the gel. PuraMatrix self-assembles when exposed to physiological levels of salt (such as culture media) into nanofibers on a scale similar to the extracellular matrix. Like collagen, PuraMatrix can be dissociated, and whilst fibre density and pore size can be more tightly controlled (a crucial criteria for drug/molecular studies), at 25% concentration necessary for encapsulation. It is the most expensive option considered here.

### Drug efficacy of 2D versus 3D varies in suspension and adherent cells

Since the primary function of most 3D cell cultures is the assessment of efficacy of new pharmaceutical interventions, it is important to understand how, and why, this response differs from a 2D culture. To this end, two drugs in wide clinical use, were investigated to benchmark their response in 3D against their 2D model.

Vinblastine is a chemo-therapeutic agent that interferes with a cell’s ability to undergo mitosis by binding to tubulin and inhibiting microtubule production causing mitotic arrest and, ultimately, cell death^30^. Amphotericin B (AmB) is an anti-fungal agent that acts by stopping the production of ergosterol which causes channels to open in the fungal cell membrane, compromising the cell and ultimately leading to cell death^31^. Both Vinblastine and Amphotericin B are small molecule drugs of similar molecular weight (909.05 Da and 924.079 Da, respectively) and as such can be assumed to diffuse similarly through hydrogels.

**Figure 4:**
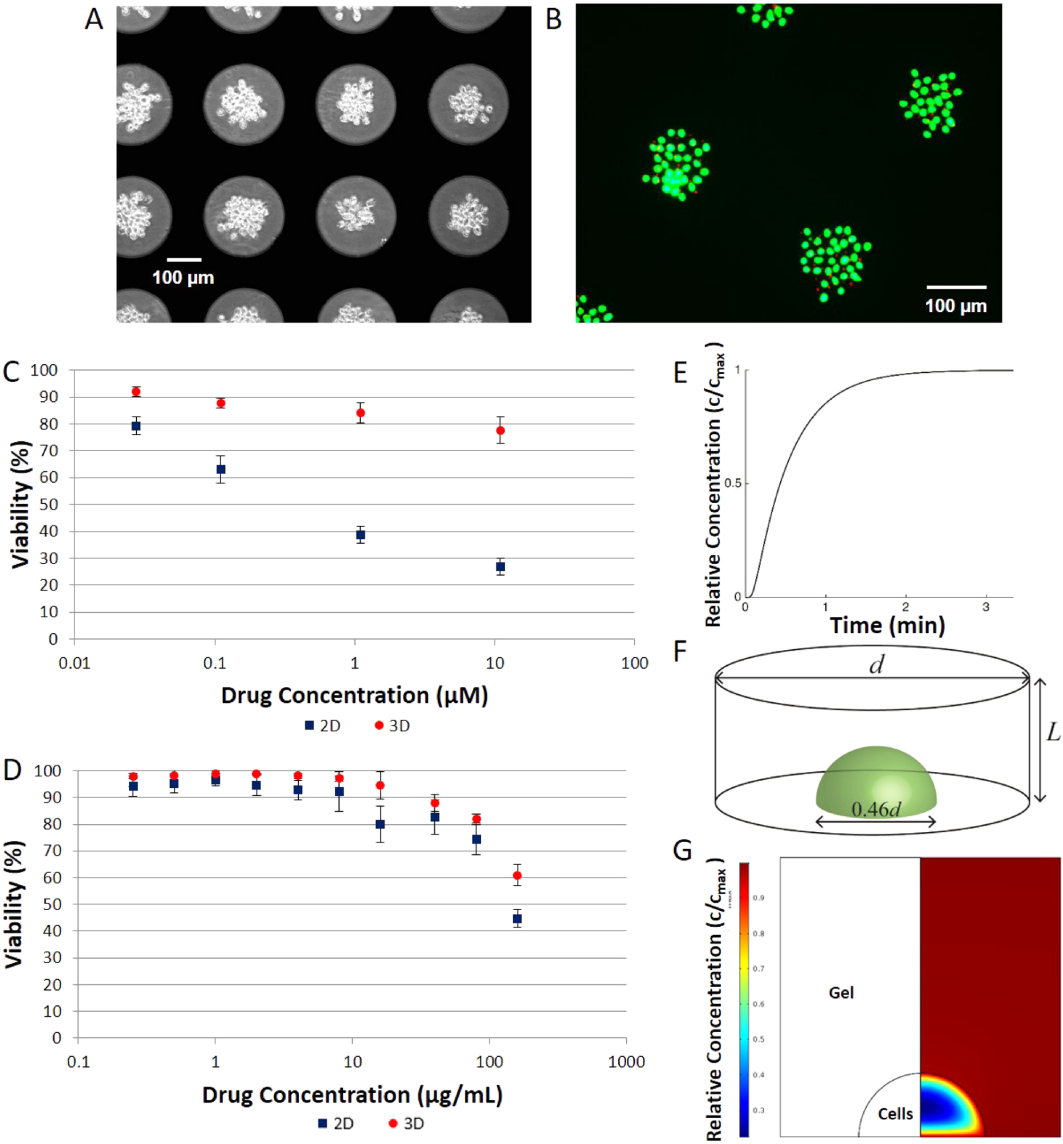
(**A**) HeLa cells aggregated prior to drug treatment and (**B**) viability of HeLa cells in aggregates assessed with live/dead assay. (**C**) Average HeLa response curves for monolayers and aggregates 48 h post-treatment with vinblastine. (**D**) Average yeast response curves for monolayers and aggregates 48 h post-treatment with Amphotericin B (**E**) Drug concentration of drug (Vinblastine/AmB) in the middle of gel layer as a fraction of the applied concentration c_max_, calculated from Eq. (3) with *D*_*g*_ = 3.3 × 10^–10^ m^2^/s and *L* = 300 µm. (**F**) Geometry of a single DEP array dot with cellular aggregate, L = 300 µm and d = 300 µm and the aggregate is modelled as a half sphere of diameter 0.46d. (**G**) Drug concentration of drug vinblastine in DEP gels as fraction of applied concentration c_max_ in a section through the centre of the cellular aggregate. Drug concentrations obtained by solving Eq. (3) in (i) a cellular aggregate region with D_c_ = 1.9 × 10^–12^ m^2^/s, k = 0.01 s^−1^ and (ii) a gel region with D_g_ = 3.3 × 10^–10^ m^2^/s and k = 0 s^−1^.

First, the effect of vinblastine on the survival of patterned and encapsulated HeLa aggregates was investigated (Fig. 4A,B). Statistical analysis indicated that the control sample for monolayer and for aggregates had no significant difference in their cell viability. The viability of monolayer cells exposed to a drug concentration of 11 μM dropped by an average of 62% after 3 h of drug incubation, compared to an average of 13% for aggregates.

When viability was measured at 48 h post treatment, it was observed to have dropped further, reaching a mean viability of 18% in 2D monolayer and 71% in 3D cell aggregates. Control samples for both 2D and 3D remained above 90% viable. The mean viability for the experiments showed that the viability for 2D monolayer dropped immediately after drug incubation, and the drug had a prolonged effect which decreased viability further 48 h post drug removal. The 3D aggregates did not show the same immediate drug effect, as viability only decreased to 78%; however, they did continue to decrease at 48 h post treatment (Fig. 4C).

This was then compared with the result of a second test; the action of AmB on yeast cells in cell aggregates. Since yeast cells are unicellular organisms which grow in suspension (in their natural shape) in isolation and do not normally interact with adjacent cells, they provide a suitable control to assess whether the process of cell resistance in clusters is due to simple blocking of cells against diffusional processes by cell packing, or a more complex process whereby cell morphology and inter-cell communication allows the cells to better adapt to the presence of the toxin. Control samples for the 2D (cells suspended in YPD broth and 3D aggregates demonstrated viability above 96%. These were also used to establish estimated growth curves of yeast over a 48 h period (due the more rapid doubling time compared to human cell lines). The treated cells started demonstrating significant viability decrease (p < 0.01) for the 2D treatment at 16 µg/ml reaching a viability below 50% at 160 µg/ml. The 3D treated cells followed a similar viability curve remaining higher than the 2D treated cells, though only significantly so (p < 0.001) for the 160 µg/ml treatment. Again, a 48 h post-treatment follow-up was conducted to examine the long-term effects of the drug treatment in 2D and 3D culture (Fig. 4D). Control samples for 2D demonstrated an 82% viability whilst the 3D samples were still 96% viable. With each increase in AmB viability decreased with the 160 µg/ml treated cells exhibiting viability of 7% and 29% for 2D and 3D, respectively.

The effectiveness of standard drug treatment of adherent cells (which naturally interact with surrounding cells in vivo) was shown to differ from that in yeast cells (which only act as independent unicellular organisms) when grown in similar conditions. The results obtained from HeLa 2D HeLa monolayers and 3D aggregates were found to be significantly different (p < 0.05) at all drug concentrations. However, the difference in drug effectiveness in suspension versus 3D culture in yeast only showed a 16% change between the different culture conditions. Taken together, this study confirms that both suspension and adherent cells do respond to drug treatments differently in a 3D environment when compared to their 2D counterpart, as has been previously demonstrated^32^. However, this also raises questions about the reason for this change in behaviour.

In order to explore this further, we developed a model of molecule diffusion through the aggregates. The drug concentrations within the entire DEP dot array can be calculated from the diffusion equation^20^. Given that the lateral extent of the array (5 mm) is significantly larger than its thickness (300 µm) it is sufficient to consider the transport of the drug through the thickness alone so that we solve:

1 $$\frac{\partial c}{{\partial t}} = D_{g} \frac{{\partial^{2} c}}{{\partial z^{2} }},$$

where $D_{g}$ is the diffusion coeffcient specific to the drug and gel. The boundary and initial conditions are $c = c_{max}$ at $z = 0$, $L$ and $c = 0$ at $t = 0$, where $c_{max}$ is the concentration of the medium in which the array is submerged and $L$ is the gel thickness, which we have taken as 300 µm throughout. The solution of Eq. (2) with these initial and boundary conditions may be obtained by the method of separation of variables as:

2 $$\frac{c}{{c_{max} }} = 1 - \mathop \sum \limits_{k = 0}^{\infty } \frac{4}{{\pi \left( {2k + 1} \right)}}\sin \left( {\frac{{\pi \left( {2k + 1} \right)z}}{L}} \right)e^{{ - \frac{{\pi^{2} \left( {2k + 1} \right)^{2} D_{g} }}{{L^{2} }}t}} .$$

A range of diffusivities have been reported in the literature^33–35^, and choosing the value corresponding to the slowest diffusion (D_g_ = 3.3 × 10^–10^ m^2^/s^27^), we plot in Fig. 4E the concentration in the middle of the DEP array. We find that the drug saturates the prepared samples within minutes. This is significantly shorter than the experimental timescales, so that the gels may be considered to be at a uniform concentration equal to the applied concentration c_max_, so that diffusion through the gel is not a limiting factor.

Turning now to a model of an individual aggregates (Fig. 4F) within each dot, we can consider each dot as a cylinder of gel with a dome-like cell aggregate at its base. The diameter of the aggregate has been measured as 0.46*d* (Table 1); in these simulations we used *d* = 300 µm (the largest value measured) and $L$ = 300 µm.

Within each dot we solved the full three-dimensional diffusion equation:

3 $$\frac{\partial c}{{\partial t}} = { }D\nabla^{2} c - f\left( c \right),$$

where $f\left( c \right)$ is a term accounting for removal of drug from the system. Within the inert encapsulating gel, we take $f\left( c \right) = 0$ and the diffusion constant $D = D_{g}$. Within the aggregates, we allow for removal of the drug from the system by cellular processes by incorporating a positive $f\left( c \right)$ and take $D = D_{c} < D_{g}$ as a modified diffusion coefficient for the cellular aggregate^36^. The boundary conditions are as before that $c = c_{max}$ at the top and bottom surfaces $z = 0$, $L$, complemented by a no-flux condition at the outer edge of the cylinder so that drug may not leave the dot through the sides. Continuity of concentration at the interface between gel and aggregate is always assumed. The initial condition is that $c = 0$ at $t = 0$.

Whilst different drug responses are often observed in 3D aggregates when compared to cells in 2D culture, it is a matter of conjecture as to whether these are due to the packed outer cells reducing the diffusion to the inner cells, or due to changes in the drug response of the individual cells when grown in 3D. Our model suggests that without any modification to drug transport within the cellular aggregates, the inner cells would be rapidly exposed to any applied small molecule drug. Indeed, our model suggests that, in the absence of an additional protection mechanism such as cellular uptake (i.e. if $\left( c \right) = 0$), uniform high concentration is achieved in time ~ O(*r*^2^/D_c_). For the small aggregates studied here, this is relatively short; simulations based on the reported diffusivities for e.g. Vinblastine in cellular aggregates^37^ show that over 90% of the cells experience $c_{max}$ within ~ 7 min. Even considering the reduced effective diffusivities that have been reported in three-dimensional tissues^33–35,38^ for a range of substances including vinblastine, oxygen, sodium fluorescein and dextrans, this is insufficient to prevent the chemicals reaching the center of the aggregate within a timescale short in comparison with the study length.

In order to account for the observed reduced effectiveness of Vinblastine in 3D we incorporated the loss term $f\left( c \right)$ in Eq. (3) when solving the diffusion equation in the aggregate. Many different functional forms for $f\left( c \right)$ are commonly used including constant^39^, linear^40^ and hyperbolic^41^. However, the data for HeLa response to vinblastine in Fig. 4C shows a relatively weak dependence on $c_{max}$, suggesting a good first-order approximation is $f\left( c \right) = - kc$; in this case the applied concentration may be scaled out of the solution by setting $c = c/c_{max}$.

This model provided a solution which demonstrated limited drug penetration to the centre of the aggregate, with the depth of drug penetration determined by the diffusive length scale parameter (α = √(*D*_*c*_*/k*)). We solved Eq. (3) (aggregate) to arrive at a steady-state solution using COMSOL (COMSOL Multiphysics v.5.2, Stockholm, Sweden). The results for vinblastine applied to HeLa aggregates (with $D_{g} = 3.3 \times 10^{ - 10}$ m^2^/s, $D_{c} = 1.9 \times 10^{ - 12}$ m^2^/s, and $k = 0.01$ s^−1^) are shown in Fig. 4G; this shows a region of low drug concentration in the centre of the aggregate which is effectively ‘protected’ by the outer layer of cells removing drug from the system by a combination of factors. Solving the full time-dependent model, we found that this steady-state solution is effectively achieved within five minutes. However, from the model alone it is impossible to distinguish between the protective effects of reducing *D*_*c*_ or increasing cellular absorption *k* as they only appear in ratio in the effective diffusive length scale.

**Figure 5:**
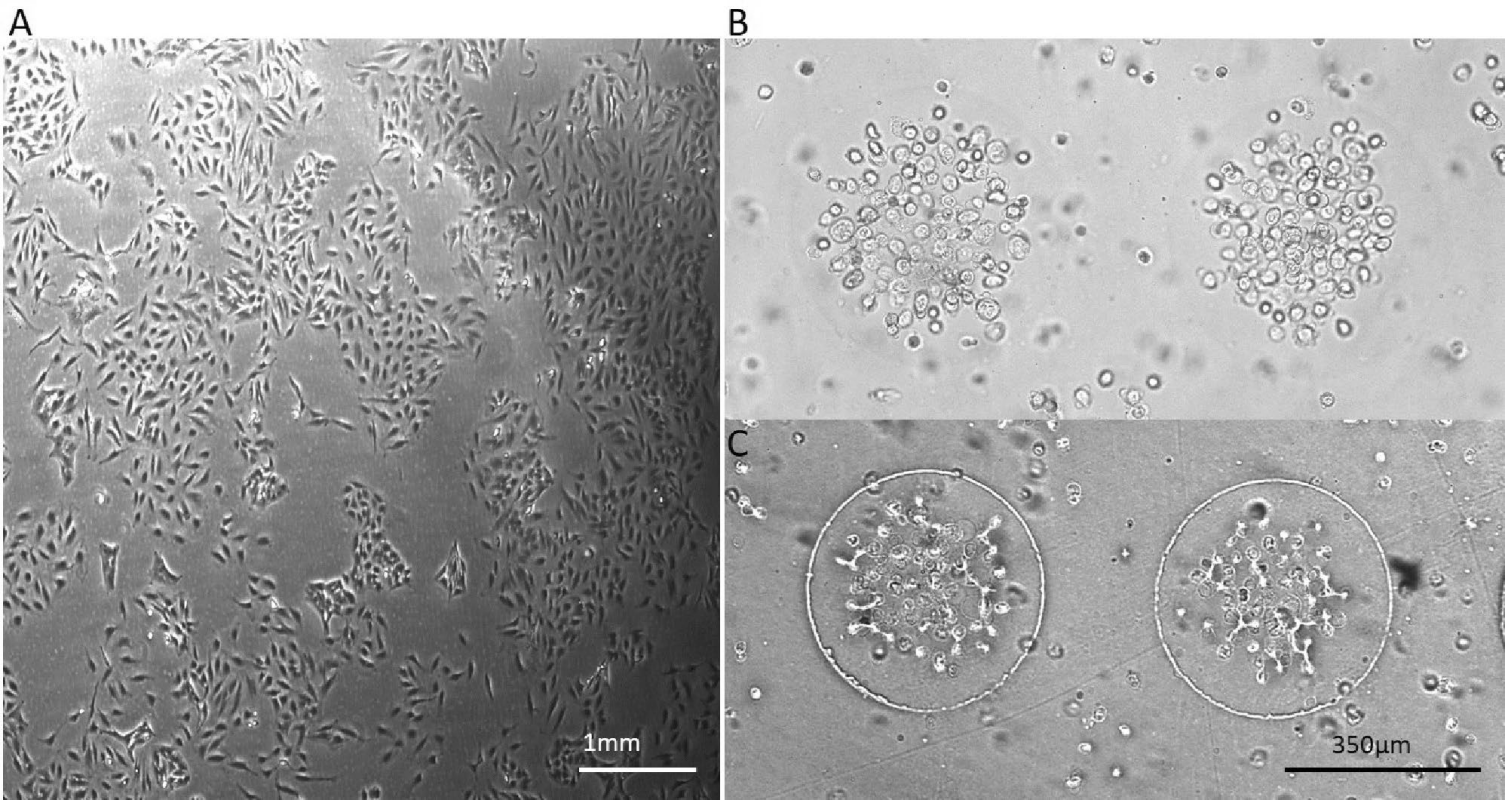
(**A**) HeLa cells grown in monolayer on a standard culture flask, (**B**) HeLa cells aggregated and 48 h post treated with 11 µM of Vinblastine and (**C**) HeLa cells aggregated and cultured with no treatment. From (**B**) it is visible that the treated cells lack the cell–cell connections shown in (**C**) of the untreated cells.

Since the tightly-packed yeast cells would present similar simple inhibitory barriers to drug diffusion in 3D to those seen in the HeLa model, we propose that this suggests that diffusion in 3D is not the primary reason for the change in HeLa behaviour, and that (as in the situation described elsewhere^36^) the primary reason for differences in cell behaviour is due indeed to cell–cell interaction and cytoplasmic changes that allow the cell to better mitigate the action of the drug in this case. In Fig. 5 HeLa cells are shown in their 2D monolayer state (Fig. 5A) in which cell attachment and actin activity can be observed, in the 3D aggregate similar cell attachment can be seen when comparing treated (non-viable) cells (Fig. 5B) to healthy cells (Fig. 5C). Compared to constructing aggregates formed spontaneously or by culturing them on treated surfaces, the hydrogel system represents a structure more like the original tissue in terms of having a polymer surrounding cells, which serves as a barrier that can represent blood (growth medium with dissolved drug) and extracellular matrix (hydrogel). Clearly this is significant in the development of new pharmaceuticals, particularly in the use of the IC50 model, where the clinical relevance of cell toxicity in vivo based on cell viability in vitro is clearly to be called into question.

### Measuring electrophysiological changes post 3D encapsulation

**Figure 6:**
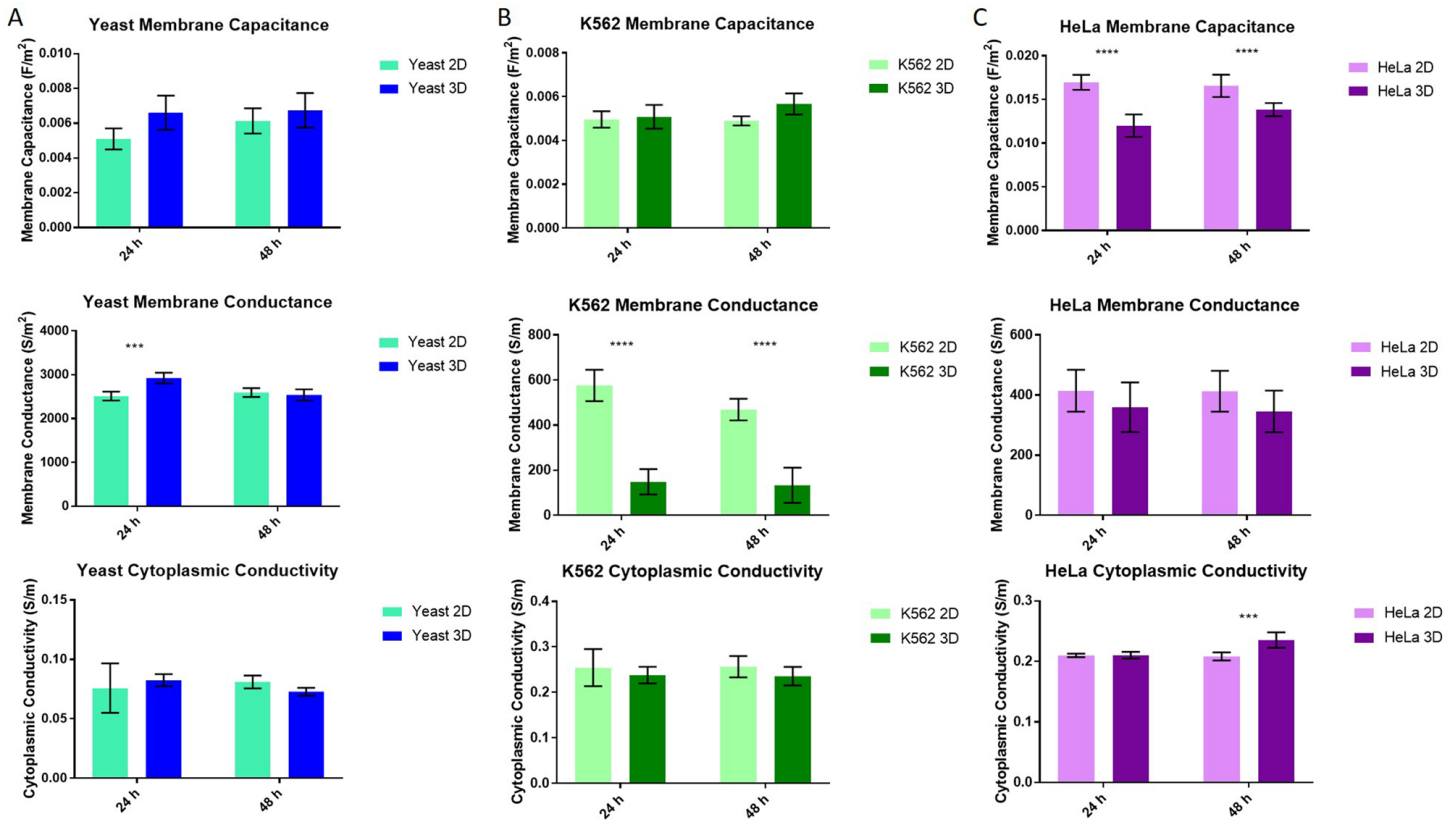
The three electrohpysiological parameters determined through the single-shell model analysis in 3DEP. Membrane capacitance, Membrane conductance, and cytoplasmic conductivity were determined for yeast (**A**), K562 (**B**) and HeLa (**C**) cells grown in their appropriate 2D models (suspension or monolayer on a flask surface) and cells removed from their 3D model formed by the DEP dot electrode using PuraMatrix gel system. Values on the graphs are given as the mean (n = 5) calculated parameter determined by fitting DEP spectra data of the cells to a single shell model ± SEM p < 0.01.

Previous work^23^ suggested that cells grown in 3D differed in their electrophysiology from those grown in 2D culture. In order to conduct a more rigorous study into the effect of DEP-based 3D cell culture on cells, we investigated the properties of yeast, K562, and HeLa cells after culture. Briefly, trypsin was added to both the 2D and 3D cell cultures for the same amount of time (this varied by a few minutes per sample, but was kept constant between the 2D and 3D replicates). Once the gels were dissociated, cells were resuspended in 10 mS/m DEP buffer, sonicated and analysed in the 3DEP reader (Labtech, Heathfield, UK)^22,29,42^. Cellular properties of cells grown in 2D for 24 h and 48 h were compared to those grown in 3D for 24 h and 48 h. The results are summarised in Fig. 6.

Analysis of the dielectric properties of these cells revealed significant (p < 0.0001) changes in the membrane capacitance for HeLa cells post 3D encapsulation compared to the 2D model (Fig. 6C) whereas for both suspension cell types, this parameter did not change significantly. Membrane conductance for both yeast and K562 demonstrated a significant change (p < 0.001) due to 3D encapsulation (Fig. 6A,B) although for yeast this effect was only observed at 24 h. The decrease in membrane conductance on the K562 cell line, with no changes in other parameters observed, could be an early indicator of changes to cell functionality^2^. In common with previous studies of cells following 3D culture^24^, variation in the membrane capacitance of the adherent HeLa cell types was the most significant change in electrophysiology post 3D encapsulation; there were no similar changes observed in the two suspension cell lines. Since no changes to cell radius were observed (n = 100 cells measured with p > 0.05 between the two groups) the change observed in the membrane capacitance of HeLa cells from 2 to 3D suggests changes to the membrane morphology have occurred.

**Figure 7:**
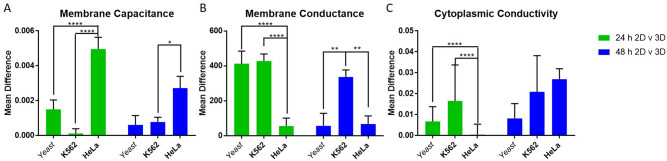
The mean difference between the 2D and 3D cell properties were determined and compared across cell types. Values given are the mean differences calculated between the means of 2D and 3D (Fig. 6) + SEM.

The mean difference between each of the properties was investigated to determine whether changes in electrophysiology between 2 and 3D were significantly different between cell types (Fig. 7). HeLa cells were found to differ between 2 and 3D in ways which differed significantly from the other two cell types (p < 0.0001 in most cases). The change in both membrane capacitance and membrane conductivity from 2 to 3D between K562 and HeLa cells was consistently significant (p < 0.0001 in most cases, p < 0.01 in Fig. 7A); interestingly, the K562 cells differed in properties in a manner similar to yeast cells (another suspension cell), rather than HeLa (another mammalian cell). Also of note is that whilst differences are still observed between 2 and 3D cells after 48 h, these differences are smaller than those observed after 24 h. It is possible that changes may be due to trypsinisation, though a study^43^ of DEP response of cells to various detachment methods suggested that this does not have a significant effect on K562 cells.

Could changes in membrane capacitance be responsible for the differences found in the 2D versus 3D drug response of HeLa cells? It is notable that the morphology change only occurs in adherent cells, and may reflect an integration of the cell layer—possibly through gap junctions—that might allow the cells to mitigate the effects of drugs. However, this would need to be investigated further.

This also demonstrates the flexibility of gel culture systems for applications that involve dissociation of the gel and aggregate for further analysis, such as analysing drug diffusion through layers of aggregate to study the necrotic core of a tumour model.

## Conclusions

The use of dielectrophoresis in the development of 3D cell aggregation for the field of tissue engineering was successfully demonstrated and optimised. This technique proved to be a robust, high-throughput, method of on-chip cell aggregation. The primary application of this technique for the use in toxicological assays was established and its utility for analysing cells post 3D encapsulation was demonstrated. There was a marked difference in the viability values in 3D cultures compared to 2D cell cultures, with adherent cell types demonstrating a greater difference in the 2D versus 3D environments than suspension cells. Analysis of the electrophysiological properties of cells cultured in 2D and 3D environments revealed changes to the membrane capacitance of the adherent HeLa cell line after 3D encapsulation was significantly different to both the yeast and K562 suspension cell types. This alludes to membrane properties, perhaps morphology or cell–cell interactions having a critical role in 3D cell drug resistance. Further, other gel systems in which aggregates can be dissociated or co-cultured demonstrated the possibility of this DEP based technique to address these critical future steps in tissue engineering.

## Methods

### DOT array fabrication

The microelectrode arrangement was developed based on the ‘DEP-dot’ system^28^; a photolithographic process^15,28^ was used to fabricate gold electrodes (EPSRC Centre for III–V Technologies, UK) of 200 nm thickness and photo-polymer resin (Poly-Diam, UK) gaskets of 300 µm thickness (Fig. 1). After thorough cleaning of the electrodes, the gasket material was temporarily adhered between the top indium tin oxide (ITO) electrode (Delta Technologies, USA) and patterned array. These were left overnight prior to aggregation.

### Continuous phase matrix (CPM)

Pre-polymer PEG-DA (MW 575) was mixed with prepared DEP medium (described for each cell type below) at 15% PEG-DA. The photo-initiator 2,2-dimethoxy-2- phenylacetophenone (DMPA) was dissolved in catalyst 1-vinyl-2-pyrrolidone (NVP) at 100 mg/ml. Once cells were resuspended in the pre-polymer DEP solution, DMPA was added to 1 ml of cell suspensions at 0.01% v/v.

We initially investigated the most commonly used blue-light photo-initiators found in the literature; camphorquinone (CQ), eosin-y (E-Y) and triethanolamine (TEA)^44^ (Sigma-Aldrich). Following protocols for optimal cell viability^37,45^ initial investigation of these initiators in the DOT electrode system revealed E-Y and TEA cured PEG in approximately 6 min, CQ and TEA cured in approximately 10 min, and CQ alone did not cure within 10 min. Thus the E-Y and TEA combination was used for all further testing. These were prepared in 15% PEG at 0.1 mM and 0.2% respectively.

Powdered gelatin (from porcine skin, type A, 300 bloom) was dissolved in a prepared buffer solution at 6.75%w/v. Conductivities of these were about 875 mS/m at 40 °C. Cell suspensions in the gelatin were kept in a water bath at 40 °C and periodically sonicated to prevent temperature dependent gelation. PuraMatrix™ was investigated at 25% concentration of the precursor solution in the prepared buffer solution with conductivity of 100 mSm^-1^.

### Yeast maintenance and preparation

Baker’s yeast (Tesco, UK) was grown in yeast extract peptone dextrose (YPD) broth (50 g/l) (Sigma Adrich, UK) at 4 ℃, collected using a sterile loupe and then grown in YPD broth for 17 h at 37 ℃. Cells were washed twice in 1 ml d-mannitol (~ 280 mmol/l) (Sigma Aldrich,UK) with PBS (Sigma Aldrich,UK) added to achieve a conductivity of 10 mS/m. Cell concentration was then adjusted to a final concentration of 2 × 10^7^ cells ml^-1^ and resuspended in prepared CPM.

### Cell line maintenance and preparation

Human K562 cells were cultured in RPMI-1640 media at 37 °C, supplemented with 10% heat-inactivated FBS (Biosera, UK), 1% l-glutamine and 1% penicillin–streptomycin. Cells were washed in low conductivity DEP buffer medium containing 8.5% sucrose and 0.3% dextrose and resuspended in CPM at a final concentration of 10^6^ cells/ml. HeLa cells were cultured in MEM with Earle’s salts and non-essential amino acids (Biosera, UK) supplemented with 10% heat-inactivated FBS (Sigma-Aldrich, UK), 1% l-glutamine and 1% penicillin–streptomycin (Sigma Aldrich, UK). At about 80% confluency, cells were trypsinized and washed twice in DEP buffer medium and resuspended in CPM at a final concentration of 10^6^ cells/ml. HL-1 cells were cultured in Claycomb media supplemented with 10% heat-inactivated FBS, 1% penicillin–streptomycin, 1% l-glutamine and Norepinephrine (0.1 mM) (Biosera, UK). The cells were trypsinised at 100% confluence and washed twice in DEP buffer medium before resuspending in CPM at a final concentration of 10^6^ cells/ml. Viability of cell suspensions was confirmed on control samples prior to experiment using a LIVE/DEAD Staining Kit (Sigma Aldrich, UK). Further viability was monitored with trypan blue (Sigma Aldrich, UK).

### DEP aggregation

For optimal negative DEP patterning, DEP crossover frequencies were established through analysis by DEPtech 3DEP reader^22,29,42^ (Labtech, Heathfield, UK) of each cell line within the CPM. DEP patterning was conducted at 10 V_pp_ and 2–10 kHz (depending on cell crossover frequency). Cell aggregation and encapsulation was carried out over 4 min (3.5 min patterning and 30 s UV exposure using the UV irradiating DEP box described elsewhere^10^). The resultant hydrogel was removed and placed in a 12 well plate with fresh culture medium. Control aggregates were assessed for viability either by trypan blue (Sigma Aldrich, UK) staining or through the LIVE/DEAD Viability assay. The hydrogels were incubated for up to 5 days depending on the purpose of the particular study.

### Hydrogel dissociation for DEP experimental

Cells were removed from their 3D model formed by the DEP dot electrode using PuraMatrix gel system for DEP experiments. This was achieved by washing the hydrogel encapsulated cells with PBS (Sigma Aldrich, UK), then pipetting tryspin (Sigma Aldrich, UK) into the well enough to cover the hydrogel layer. Simultaneously, trypsin was added to a culture flask of 2D cultured cells washed with PBS. Time was monitored to ensure 3D and 2D tryspin exposure remained consistent. When necessary, sonication of the aggregates was performed to break apart clumps of cells. Once cells were dissociated, cells were washed in 5 ml of their respective DEP buffer and prepared at a concentration of 10^6^ cells/ml for DEP characterization.

### Drug treatment and measurement

#### Yeast treatment

Amphotericin B (AmB), a common anti-fungal drug, was prepared by dissolving 5 mg of AmB powder in 1 ml of DMSO and then mixed in YPD broth for a final concentration of 160.0 µg/ml. Serial dilutions were then used to make up concentrations ranging from 5 to 160 µg/ml which, when applied in a 10:1 dilution gave applied concentrations ranging from 0.5 to 16 µg/ml. A 12-well plate was used to conduct drug tests on both 2D cell suspensions and 3D aggregates. Cells encapsulated in hydrogels followed the same drug treatment regime as those in YPD solution.

#### HeLa treatment

Vinblastine sulphate salt powder ≥ 96% (Sigma Aldrich, UK), a common anti-cancer drug, was dissolved in sterile filtered distilled water (10 mg/ml) to make up a stock solution of 11 mM. For each experiment 100 µl of stock solution was diluted in complete HeLa growth medium to make up four different concentrations of 11 μM, 1.1 μM, 0.1 μM and 27.5 nM, selected based on previous work^1,46^ and henceforth referred to as concentrations 1–4, respectively. Hydrogels were kept in well plates containing complete growth medium solution for 5 days (the time at which cells were observed to begin adhering to one another). The drug was administered and left to incubate for 3 h. Viability was tested using Trypan blue prior to and after treatment.

For 2D monolayer experiments, six T75 flasks were used to seed HeLa cells taken from the same source used to make each set of hydrogels. In order to use the same cell sample through the treatment no staining was administered to assess viability. Instead, for each drug concentration cell counts were taken of adherent cells on defined areas of the flask prior to drug treatment. Cells were treated and then incubated for 3 h. The same defined area of the flask was then counted for adherent cells. Following this, both monolayers and hydrogels were incubated for 48 h to examine any effects of post vinblastine treatment.
